# Conditionally Immortalized Mouse Embryonic Fibroblasts Retain Proliferative Activity without Compromising Multipotent Differentiation Potential

**DOI:** 10.1371/journal.pone.0032428

**Published:** 2012-02-23

**Authors:** Enyi Huang, Yang Bi, Wei Jiang, Xiaoji Luo, Ke Yang, Jian-Li Gao, Yanhong Gao, Qing Luo, Qiong Shi, Stephanie H. Kim, Xing Liu, Mi Li, Ning Hu, Hong Liu, Jing Cui, Wenwen Zhang, Ruidong Li, Xiang Chen, Jikun Shen, Yuhan Kong, Jiye Zhang, Jinhua Wang, Jinyong Luo, Bai-Cheng He, Huicong Wang, Russell R. Reid, Hue H. Luu, Rex C. Haydon, Li Yang, Tong-Chuan He

**Affiliations:** 1 School of Bioengineering, Chongqing University, Chongqing, China; 2 Molecular Oncology Laboratory, Department of Surgery, The University of Chicago Medical Center, Chicago, Illinois, United States of America; 3 Stem Cell Biology and Therapy Laboratory of the Key Laboratory for Pediatrics co-designated by Chinese Ministry of Education and Chongqing Bureau of Education, The Children's Hospital of Chongqing Medical University, Chongqing, China; 4 Key Laboratory of Diagnostic Medicine designated by the Chinese Ministry of Education, and the Affiliated Hospitals of Chongqing Medical University, Chongqing, China; 5 Department of Cell Biology, Third Military Medical University, Chongqing, China; 6 Institute of Materia Medica, Zhejiang Chinese Medical University, Hangzhou, China; 7 Department of Geriatrics, Xinhua Hospital of Shanghai Jiatong University, Shanghai, China; 8 Department of Orthopaedic Surgery, The Affiliated Tangdu Hospital, Fourth Military Medical University, Xi'an, China; University of Medicine and Dentistry of New Jersey, United States of America

## Abstract

Mesenchymal stem cells (MSCs) are multipotent cells which reside in many tissues and can give rise to multiple lineages including bone, cartilage and adipose. Although MSCs have attracted significant attention for basic and translational research, primary MSCs have limited life span in culture which hampers MSCs' broader applications. Here, we investigate if mouse mesenchymal progenitors can be conditionally immortalized with SV40 large T antigen and maintain long-term cell proliferation without compromising their multipotency. Using the system which expresses SV40 large T antigen flanked with Cre/loxP sites, we demonstrate that mouse embryonic fibroblasts (MEFs) can be efficiently immortalized by SV40 large T antigen. The conditionally immortalized MEFs (iMEFs) exhibit an enhanced proliferative activity and maintain long-term cell proliferation, which can be reversed by Cre recombinase. The iMEFs express most MSC markers and retain multipotency as they can differentiate into osteogenic, chondrogenic and adipogenic lineages under appropriate differentiation conditions *in vitro* and *in vivo*. The removal of SV40 large T reduces the differentiation potential of iMEFs possibly due to the decreased progenitor expansion. Furthermore, the iMEFs are apparently not tumorigenic when they are subcutaneously injected into athymic nude mice. Thus, the conditionally immortalized iMEFs not only maintain long-term cell proliferation but also retain the ability to differentiate into multiple lineages. Our results suggest that the reversible immortalization strategy using SV40 large T antigen may be an efficient and safe approach to establishing long-term cell culture of primary mesenchymal progenitors for basic and translational research, as well as for potential clinical applications.

## Introduction

Mesenchymal stem cells (MSCs) are non-hematopoietic multipotent cells, which have the capacity to differentiate into tissues of both mesenchymal and non-mesenchymal origin [1]–[6]. MSCs can differentiate into osteoblastic, chondrogenic, and adipogenic lineages [1]–[6], although it has recently been reported that MSCs are also able to differentiate into other lineages, including neuronal [7]–[9] and cardiomyogenic [10] lineages. Unlike hematopoietic or neuronal stem cells, MSCs usually lack specific markers. Nonetheless, it is generally accepted that MSCs have to satisfy three criteria [11]. First, MSCs must be plastic-adherent when maintained in standard culture conditions using tissue culture flasks. Second, more than 95% of the MSC population must express CD105, CD73 and CD90, but must lack expression (<2% positive) of CD45 and CD34. Third, the cells must be able to differentiate to osteoblasts, adipocytes and chondroblasts under standard *in vitro* differentiating conditions [11].

MSCs have attracted significant attention for their potential role in elucidating stem differentiation pathways, promoting tissue engineering for regenerative medicine, and functioning as gene vectors and immunomodulators in autoimmune diseases [5], [6], [12]–[16]. In addition to bone marrow, MSCs have been isolated from almost every type of tissue, including periosteum, brain, liver, bone marrow, adipose, skeletal muscle, amniotic fluid and hair follicle [17]–[26]. While MSCs isolated from various tissues share many similar characteristics, they exhibit minor differences in their expression profile and differentiation potential [27].

One of the major technical challenges is to isolate sufficient MSCs for *in vitro* and *in vivo* studies, as well as to expand MSCs for possible clinical applications. For example, in bone marrow MSCs only make up a minute fraction of nucleated cells and account for approximately 0.001%–0.01% of all cells in each aspirate, depending on the technique [2]. The therapeutic application of MSCs would need an even large number of cells, which requires *ex vivo* expansion post-harvest [5], [6], [12].

In this study, we sought to investigate if conditionally immortalized mesenchymal progenitor cells can maintain long-term cell proliferation without compromising the multipotent differentiation potential. Technologically, we take advantage of the previously characterized reversible immortalization system, which expresses SV40 T antigen flanked with Cre/loxP sites [28]. We have demonstrated that mouse embryonic fibroblasts (MEFs) can be effectively immortalized with an enhanced proliferative activity. The immortalized MEFs (iMEFs) express most of the MSC markers (CD90/Thy-1^+^, CD73^+^, CD105/Endoglin^+^, CD166/ALCAM^+^, and CD44^+^), suggesting that the iMEFs may be MSC-like. We previously demonstrated that BMP9 (aka, growth and differentiation factor 2, or GDF2) is one of the most potent factors that can induce osteogenic and adipogenic, to a lesser extent, chondrogenic differentiation [29]–[33]. We demonstrate that BMP9 can up-regulate the key lineage-specific regulators Runx2 (osteogenic), Sox9 (chondrogenic) and PPARγ2 (adipogenic), and that BMP9 can induce osteogenic marker alkaline phosphatase activity (ALP) and matrix mineralization in the iMEFs *in vitro*. Moreover, the iMEFs are able to undergo terminal adipogenic differentiation upon BMP9 or PPARγ2 stimulation. Introduction of Cre recombinase into the iMEFs effectively removes SV40 large T antigen and results in a significant decrease in cell proliferation. Furthermore, *in vivo* stem cell implantation studies indicate that the iMEFs are able to bone, cartilage and adipose tissues upon BMP9 stimulation, whereas in the presence of Cre recombinase the ability of iMEFs to differentiate into these tissues is decreased possibly due to the reduced expansion of progenitor population. Lastly, we demonstrate that the iMEFs fail to form any subcutaneous tumors when injected into athymic nude mice for up to 10 weeks. Taken together, our results have demonstrated that the conditionally immortalized iMEFs not only maintain long-term cell proliferation but also retain the ability to differentiate into multiple lineages. The reversible SV40 T-mediated immortalization strategy should be used to establish stable cells from primary progenitors isolated from limited tissue sources, which may be critical for basic and translational studies.

## Materials and Methods

### Cell culture and chemicals

Human bone marrow stromal stem cells were purchased from ScienCell Research Laboratories (Carlsbad, CA). Human osteosarcoma line 143B and HEK-293 cells were from ATCC (Manassas, VA). The cells were maintained in the completed DMEM medium described [29], [34]–[37]. Unless indicated otherwise, all chemicals were purchased from Sigma-Aldrich or Fisher Scientific.

### Isolation of mouse embryo fibroblasts (MEFs) and establishment of reversibly immortalized MEFs (iMEFs)

MEFs were isolated from post coitus day 12.5–13.5 CD1 mice as previously described [37]–[39]. Each embryo was dissected into 10 ml sterile PBS, voided of its internal organs, and sheared through an 18-gauge syringe in the presence of 1 ml 0.25% trypsin and 1 mM EDTA. After 15 min incubation with gentle shaking at 37°C, DMEM with 10% fetal bovine serum (FBS) was added to inactivate trypsin. The cells were plated on 100 mm dishes and incubated for 24 h at 37°C. Adherent cells were used as MEF cells. Aliquots were kept in a liquid nitrogen tank. All MEFs used in this study were within five passages.

**Figure 1:**
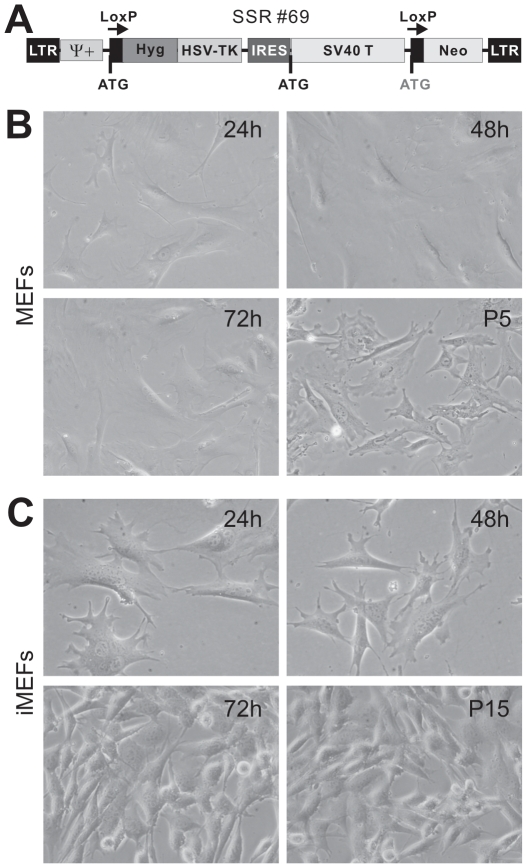
Morphology of primary and immortalized mouse embryonic fibroblasts (MEFs). (**A**) Schematic representation of the reversible immortalization vector SSR #69 [28]. This retroviral vector contains the hygromycin and SV40 T antigen expression cassette flanked by loxP sites. (**B**) Morphology of primary MEFs in cell culture. Primary MEFs were seeded at 20–30% confluence and passed consecutively for five passages (P5). (**C**) Morphology of the reversibly immortalized MEFs (iMEFs) in cell culture. The iMEF cells were seeded at low density and passed consecutively for 15 passages (P15).

To establish the immortalized MEFs (iMEFs), early passage MEFs (<3 passages) were seeded in 25 cm^2^ flasks and infected with packaged retrovirus SSR #69, which expresses SV40 T Ag flanked with Cre/loxP sites (**Fig. 1A**) [28]. Stable iMEF cell pools were established by selecting the infected cells with hygromycin B (at 4 µg/ml) for one week. Aliquots of the iMEFs were kept in liquid nitrogen tanks. Human bone marrow stromal stem cells were immortalized in a similar fashion.

### Recombinant adenoviruses expressing BMP9, PPARγ2, Cre and GFP

Recombinant adenoviruses were generated using the AdEasy technology as described [29], [30], [32], [40]–[42]. The coding regions of human BMP9, mouse PPARγ2, Cre recombinase and green fluorescence protein (GFP) were PCR amplified and cloned into an adenoviral shuttle vector and subsequently used to generate recombinant adenoviruses in HEK293 cells. The resulting adenoviruses were designated as AdBMP9, AdPPARγ2 and Ad-Cre, which also express GFP as a marker for monitoring infection efficiency. Analogous adenovirus expressing only GFP (AdGFP) was used as controls [37], [38], [40]–[47].

### RNA isolation and semi-quantitative RT-PCR analysis

Total RNA was isolated using TRIZOL Reagents (Invitrogen). Total RNA was used to generate cDNA templates by RT reaction with hexamer and M-MuLV Reverse Transcriptase (New England Biolabs, Ipswich, MA). The first strand cDNA products were further diluted 5- to 10-fold and used as PCR templates. Semi-quantitative RT-PCR was carried out as described [32], [34], [37], [38], [47]–[50]. PCR primers for mouse Runx2 (5′-CCG GTC TCC TTC CAG GAT-3′ and 5′-GGG AAC TGC TGT GGC TTC-3′), Sox9 5′-AGC TCA CCA GAC CCT GAG AA-3′ and 5′-TCC CAG CAA TCG TTA CCT TC-3′), PPARγ2 5′-ACT GCC GGA TCC ACA AAA-3′ and 5′-TCT CCT TCT CGG CCT GTG-3′) transcripts were designed by using *Primer3 Plus* program (http://www.bioinformatics.nl/cgi-bin/primer3plus/primer3plus.cgi) to amplify the genes of interest (approximately 150–180 bp). A touchdown cycling program was as follows: 94°C for 2 min for 1 cycle; 92°C for 20 s, 68°C for 30 s, and 72°C for 12 cycles with a decrease in 1°C per cycle; and then at 92°C for 20 s, 57°C for 30 s, and 72°C for 20 s for 20–25 cycles, depending on the abundance of a given gene. The specificity of PCR products were confirmed by resolving PCR products on 1.5% agarose gels. All samples were normalized by the expression level of GAPDH.

### Alkaline phosphatase (ALP) activity assay

ALP activity was assessed quantitatively with a modified assay using the Great Escape SEAP Chemiluminescence assay kit (BD Clontech, Mountain View, CA) and qualitatively with histochemical staining assay (using a mixture of 0.1 mg/ml napthol AS-MX phosphate and 0.6 mg/ml Fast Blue BB salt) as described [29], [30], [32], [34], [37]–[39], [45]–[47]. Each assay condition was performed in triplicate and the results were repeated in at least three independent experiments. ALP activity was normalized by total cellular protein concentrations among the samples.

### Matrix mineralization assay (Alizarin Red S staining)

MEFs were seeded in 24-well cell culture plates and infected with AdBMP9 or AdGFP. Infected cells were cultured in the presence of ascorbic acid (50 µg/mL) and β-glycerophosphate (10 mM). At 14 days after infection, mineralized matrix nodules were stained for calcium precipitation by means of Alizarin Red S staining as described [29], [30], [32], [34], [37]–[39], [45]–[47]. Cells were fixed with 0.05% (v/v) glutaraldehyde at room temperature for 10 min and washed with distilled water, fixed cells were incubated with 0.4% Alizarin Red S (Sigma-Aldrich, St. Louis, MO) for 5 min, followed by extensive washing with distilled water. The staining of calcium mineral deposits was recorded under bright field microscopy.

### Immunofluorescence staining

Immunofluorescence staining was performed as described [32], [34], [37], [48]–[50]. Briefly, cells were fixed with methanol, permeabilized with 1% NP-40, and blocked with 10% BSA, followed by incubating with CD73, CD44, CD90, CD117/c-kit, CD29, CD133, CD105/endoglin, CD166/ALCAM, or BMPR-II antibody (Santa Cruz Biotechnology) for 1 h. After washing, cells were incubated with Texas Red-labeled secondary antibody (Santa Cruz Biotechnology) for 30 min. Cell nuclei were stained with DAPI. Stains were examined under a fluorescence microscope. Stains without primary antibodies, or with control IgG, were used as negative controls.

### Immunohistochemical staining

Cultured cells were infected with adenoviruses. At the indicated time points, cells were fixed with 10% formalin and washed with PBS. The fixed cells were permeabilized with 1% NP-40 and blocked with 10% goat serum, followed by incubation with an anti-osteocalcin, or osteopontin antibody (Santa Cruz Biotechnology) for 1 h. After washing, cells were incubated with biotin-labeled secondary antibody for 30 min, followed by incubating cells with streptavidin-HRP conjugate for 20 min at room temperature. The presence of the expected protein was visualized by DAB staining and examined under a microscope. Stains without the primary antibody, or with control IgG, were used as negative controls.

### Western blotting analysis

Western blotting was performed as previously described [37], [39], [47]–[52]. Briefly, cells were collected in Lysis Buffer. Cleared total cell lysate was denatured by boiling and loaded onto a 10% gradient SDS–PAGE. After electrophoretic separation, proteins were transferred to an Immobilon-P membrane. Membrane was blocked with SuperBlock Blocking Buffer, and probed with the primary antibodies, anti-SV40 T Ag or anti-β-actin (Santa Cruz Biotechnology, Santa Cruz, CA), followed by incubation with a secondary antibody conjugated with horseradish peroxidase. The proteins of interest were detected by using SuperSignal West Pico Chemiluminescent Substrate kit.

### Subcutaneous stem cell implantation

All animal work was conducted according to relevant national and international guidelines. The animal welfare, use, and care were carried out according to the approved Institutional Animal Care and Use Committee (IACUC) of The University of Chicago (protocol # 71108). Stem cell-mediated ectopic bone formation was done as described [29], [30], [32], [37]–[39]. Briefly, MEFs cells were infected with AdBMP9 or AdRFP for 16 h, collected and resuspended in PBS for subcutaneous injection (5×10^6^/injection) into the flanks of athymic nude (nu/nu) mice (5 animals per group, 4–6 wk old, female, Harlan Sprague-Dawley). At 4 wk after implantation, animals were sacrificed, and the implantation sites were retrieved for histologic evaluation, and other specialty stains.

### Xenogen bioluminescence imaging of iMEF subcutaneous injection

The iMEFs were stably labeled with firefly luciferase and injected into the flanks of anythmic nude mice (2×10^6^ cells/injection; n = 6, 4–6 wk old, female, Harlan Sprague-Dawley) [34], [42], [51], [53]. At the indicated time points, animal were anesthetized with isoflurane attached to a nosecone mask within Xenogen IVIS 200 imaging system. For bioluminescence imaging, animals were injected (i.p.) with D-Luciferin sodium salt (Gold BioTechnology) at 100 mg/kg in 0.1 ml sterile saline. Pseudoimages were obtained by superimposing the emitted light over the gray-scale photographs of the animals.

### Hematoxylin & eosin, trichrome, and alcian blue staining

Retrieved tissues were fixed in 10% formalin overnight and embedded in paraffin. Serial sections of the embedded specimens were stained with hematoxylin and eosin (H & E). Trichrome and alcian blue stains were carried out as previously described [29], [30], [32].

## Results

### Immortalized mouse embryonic fibroblasts (iMESs) exhibit high proliferative activity

Numerous studies have been recently carried out to induce the pluripotency of fibroblasts and MEFs through reprogramming stem cell differentiation capability with a set of defined factors, such as Oct3/4, Sox9, Klf4, and c-Myc [54]–[57]. We sought to investigate whether immortalized MEFs retain the multipotent properties of mesenchymal stem cells (MSCs) by taking advantage of a previously characterized reversible immortalization system using SV40 T antigen (**Fig. 1A**) [28]. The reversible immortalization vector SSR #69 contains the hygromycin and SV40 T antigen expression cassette flanked with loxP sites [28]. Primary MEFs were shown to grow fairly well, albeit at lower rate, up to at least five passages (**Fig. 1B**). The immortalized MEFs (iMEFs) grew more rapidly and maintained a high proliferation rate after 15 passages (**Fig. 1C**). In fact, iMEFs have been passed more than 50 generations by now and proliferate well. Thus, these results indicate that we successfully immortalized MEFs *in vitro*.

**Figure 2:**
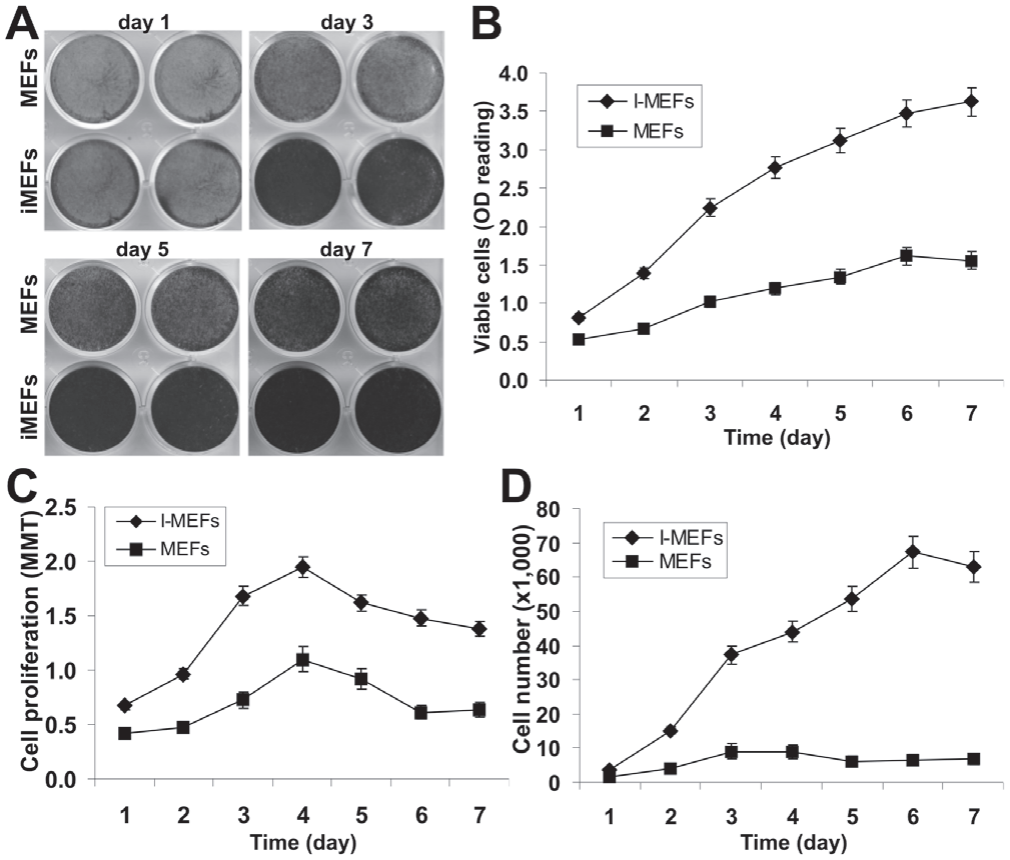
The iMEFs exhibit higher proliferative activity than that of primary MEFs. (**A**) and (**B**) Cell viability and proliferation assay by crystal violet staining assay. The same number of primary MEF and iMEF cells was seeded at a low density. Cells were stained with crystal violet at the indicated time points (A) and the viable and stained cells were dissolved for OD reading as previously described (B) [42]. (**C**) Cell proliferation assessed with MTT assay. The same cell number of primary MEFs and iMEFs was seeded at a low density. Cells were collected for MTT assay at the indicated time points. (**D**) Cell counting assay. The same number of primary MEF and iMEF cells was seeded at 20% confluence. Cells were trypsinized, stained with Trypan blue, and counted at the indicated time points. For all of the above assays, each assay condition was done in triplicate. The assays were repeated in at least two independent batches. Representative results are shown.

Next, we quantitatively compared the proliferative activity between primary MEFs and iMEFs. Crystal violet staining assay indicated that iMEF cells reached complete confluence at day 3 while MEF cells reached complete confluence at day 5 when both started with a similar cell density (**Fig. 2A**). A quantitative assessment of the stained cells confirmed the staining results and revealed that iMEFs had significantly higher cell staining at each given time points than that of the MEFs (**Fig. 2B**). The iMEF cells exhibited a higher proliferation rate than that of MEFs in MTT assay when the same number of primary MEF and iMEF cells was seeded at a low density (**Fig. 2C**). Direct cell counting experiment further confirmed that iMEF cells grow faster than MEFs (**Fig. 2D**). Taken together, these results demonstrate that immortalized MEFs can be maintained in culture and exhibit much higher proliferation rate.

### iMEFs express MSC markers

**Figure 3:**
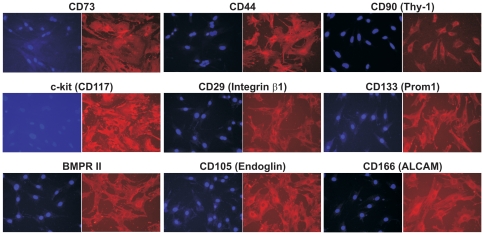
The iMEFs express most mesenchymal stem cell markers. The iMEF cells were seeded at subconfluency for 24 h and stained with MSC marker antibodies as indicated. The consensus MSC markers include CD44, CD90/Thy-1, CD73, CD105/Endoglin and CD166/ALCAM [11]. Cell nuclei were stained with DAPI. Respective IgG Isotypes were used as immunostaining control.

To assert if iMEFs possess MSC-like multipotency, we characterized the expression of MSC markers using immunofluorescence staining. It has been reported that the consensus human MSC markers include CD90/Thy-1, CD73, CD105/Endoglin, CD166/ALCAM and CD44 [11]. Although mouse MSCs do not necessarily express all the same molecules as those on human cells, we found that all of these markers were readily detectable in iMEFs by immunofluorescence staining (**Fig. 3**). Flow cytometry indicated that more than 90% of MSC population expresses these markers (data not shown). Furthermore, we found that the expression of CD45 and CD34 was not detectable (data not shown). Thus, these results demonstrate that the iMEFs express most if not all of the conventional MSC markers, suggesting that these cells may possess MSC-like phenotypes.

### iMEF cells are capable of differentiating into osteogenic, chondrogenic, and adipogenic lineages

**Figure 4:**
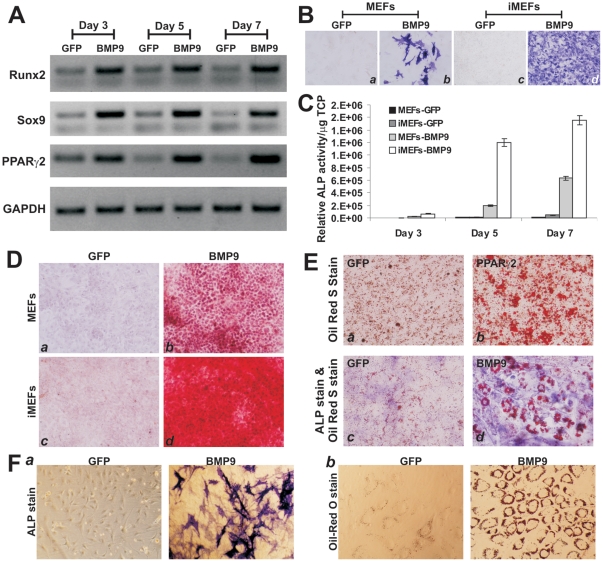
Induction of osteogenic, chondrogenic, and adipogenic lineage markers in iMEFs. (**A**) Expression of lineage-specific regulators in iMEFs stimulated by BMP9. Subconfluent iMEF cells were infected with AdBMP9 or AdGFP. Total RNA was isolated at the indicated time points and subjected to RT-PCR reactions. The cDNA products were used as templates for semi-quantitative amplification of mouse Runx2, Sox9 and PPARγ2 transcripts. All samples were normalized with GAPDH expression levels. (**B**) and (**C**) Induction of early osteogenic marker alkaline phosphatase (ALP) in primary MEFs and iMEFs. Subconfluent primary MEFs and iMEFs were infected with AdBMP9 or AdGFP. ALP activity was histochemically stained on day 5 (B) or quantitatively determined at days 3, 5 and 7 (C). The ALP activity was normalized with total cellular protein (TCP) (C). (**D**) Matrix mineralization assessed with Alizarin Red S staining. Subconfluent MEFs and iMEFs were infected with AdBMP9 or AdGFP for 14 days. Cells were fixed and stained with Alizarin Red S. (**E**) Adipogenic differentiation assessed with Oil Red O staining. Subconfluent iMEFs were infected with AdBMP9, AdPPARγ2, or AdGFP for 10 days. Cells were fixed and stained with Oil Red O staining (*panels a and b*), or stained for ALP activity, followed by Oil-Red O staining (*panels c and d*). (**F**) Osteogenic and adipogenic differentiation of immortalized human bone marrow stromal stem cells. Subconfluent cells were infected with AdBMP9 or AdGFP. ALP staining was carried out at day 7 (*a*) while Oil-red O staining was done at day 14 (*b*). Each assay condition was done in triplicate. The assays were repeated in at least two independent batches. Representative results are shown.

Given the fact that MSC markers are not specific and unique, MSCs are by definition required to give rise to at least osteogenic, chondrogenic and adipogenic lineages [1]–[5]. We tested if the iMEFs were able to differentiate into these lineages. We have demonstrated that BMP9 is one of the most potent factors that can induce osteogenic and adipogenic, to a lesser extent, chondrogenic differentiation [29]–[33]. When the iMEFs were transduced with BMP9 or GFP adenoviral vector, the three key lineage-specific regulators, Runx2 (osteogenic), Sox9 (chondrogenic) and PPARγ2 (adipogenic), were significantly up-regulated at as early as day 3 (**Fig. 4A**). We further analyzed the early osteogenic marker alkaline phosphatase (ALP) in these cells. As shown in **Fig. 4B**, BMP9 induced much higher ALP activity at day 5 in the iMEFs than that in primary MEFs. The ALP activity induced by BMP9 in iMEFs and MEFs was also confirmed quantitatively (**Fig. 4C**). The BMP9-transduced iMEFs were further shown to effectively undergo late stage of osteogenic differentiation as evidenced by matrix mineralization assessed with Alizarin Red S staining (**Fig. 4D**).

We next analyzed the adipogenic potential of iMEFs. In addition to BMP9, PPARγ2 has been shown to induce adipogenic differentiation of MSCs [32]. When the iMEFs were transduced with PPARγ2 adenoviral vector, a significant portion of the cells underwent terminal adipogenic differentiation as evidenced by Oil Red-O staining (**Fig. 4E**, panels **a** and **b**). Furthermore, when iMEF cells were stimulated with BMP9 for 10 days, a double staining of the transduced cells for ALP activity and Oil Red-O positivity revealed that iMEFs stimulated by BMP9 can undergo either osteogenic or adipogenic differentiation, a process was shown to be mutually exclusive (**Fig. 4E**, panels **c** and **d**). These results are consistent with what we previously reported in MSCs [29]–[33]. Furthermore, using the same procedure, we immortalized human bone marrow stromal stem cells and found that the immortalized bone marrow stromal cells also acquired long-term proliferative capability but retained the ability to undergo osteogenic (**Fig. 4F**, panel **a**) and adipogenic potential (**Fig. 4F**, panel **b**) upon BMP9 stimulation. Taking the in vitro results together, we have demonstrated that the iMEFs are multipotent and capable of differentiating into osteogenic, chondrogenic and adipogenic lineages.

### The enhanced proliferative activity of the iMEFs can be reversed by Cre recombinase

**Figure 5:**
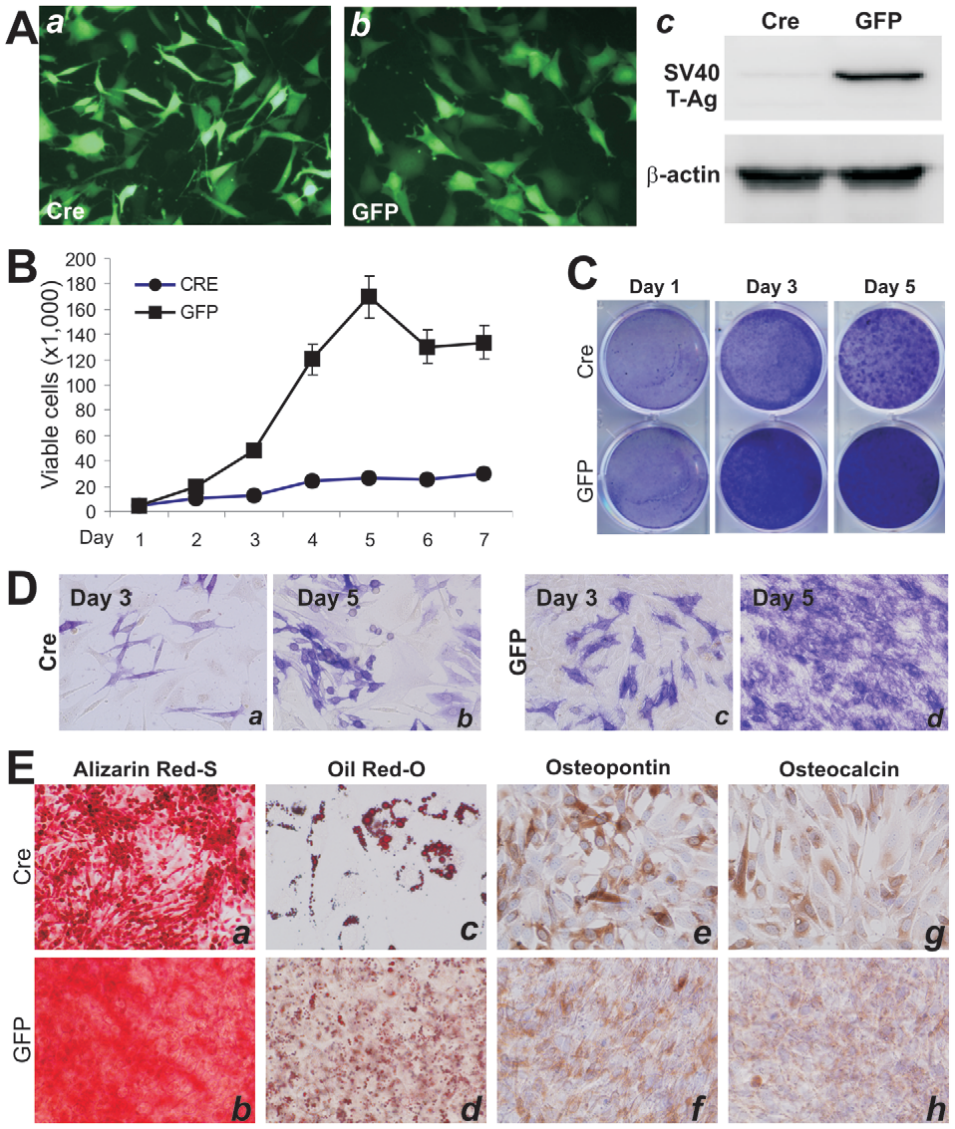
The proliferation properties of the iMEFs can be reversed by Cre. (**A**) Efficient transduction of iMEFs by adenoviral vectors. Subconfluent iMEFs were infected with AdCre (*a*) or AdGFP (*b*) for 24 h. GFP signal was recorded under fluorescence microscopy. (**c**) Cre-mediated efficient removal of SV40 T Ag detected by Western blotting. Subconfluent iMEFs were infected with AdCre or AdGFP for 36 h. Cells were lysed, and cell lysate was subjected to Western blotting using anti-SV40 T Ag antibody. Anti-ß-actin Western blotting confirms equal loading of the samples. (**B**) Cell proliferation by viable cell counting. AdCre or AdGFP-transduced iMEFs were counted at indicated time points as described in **Figure 2D**. (**C**) Cell proliferation assessed by crystal violet staining. AdCre or AdGFP-transduced iMEFs were stained with crystal violet at indicated time points as described in **Figure 2A**. (**D**) Effect on ALP activity in iMEFs by Cre. AdCre or AdGFP-transduced iMEFs were stained for ALP activity at indicated time points. (**E**) The effect of Cre-mediated reversal on late stage differentiation of the iMEFs. Subconfluent iMEFs were infected with AdCre or AdGFP and maintained for 14 days. Cells were processed with Alizarin Red S staining (*a and b*) and Oil Red-O staining (*c and d*), or immunostained against osteogenic late markers osteopontin (*e and f*) and osteocalcin (*g and h*). See Methods.

We sought to text if the immortalization-related phenotypes of the iMEFs could be reversed. As shown in **Fig. 1A**, the immortalizing gene SV40 large T antigen could be removed through the action of Cre recombinase on the flanking loxP sites. To effectively express Cre in the iMEFs, we constructed a recombinant adenoviral vector AdCre, which was shown to transduce iMEFs with high efficiency (**Fig. 5A**, panels **a** & **b**). The efficient removal of SV40 T antigen by Cre expression was shown using anti-T antigen Western blotting in AdCre infected iMEFs, but not in the GFP control (**Fig. 5A**, panel **c**). Cell proliferation rate of the AdCre-transduced iMEFs was significantly decreased in both cell counting (**Fig. 5B**) and Crystal violet staining (**Fig. 5C**) assays. Thus, these results indicate that the proliferative activity of the iMEFs can be effectively reversed by Cre recombinase.

We further analyzed the differentiation potential of the Cre-transduced iMEFs. When iMEFs were infected with AdCre or AdGFP in the presence of BMP9, the ALP activity was induced in Cre-transduced iMEFs, albeit to a much lower extent than that of the GFP controls (**Fig. 5D**). The effect of Cre-mediated reversal on late stage differentiation of the iMEFs was also examined. When subconfluent iMEFs were infected with AdCre or AdGFP for 14 days, cells were stained with Alizarin Red S staining (**Fig. 5E** panels a & b) and Oil Red-O staining (**Fig. 5E** panels c and d), or immunostained against osteogenic late markers osteopontin (**Fig. 5E** panels e and f) and osteocalcin (**Fig. 5E** panels g and h). In these assays, Cre-tranduced iMEFs were still able to differentiate into osteogenic and adipogenic lineages, which were significantly less effective than the GFP controls. Thus, these results strongly suggest that Cre-mediated reversal of the immortalization phenotypes may significantly reduce the differentiation potential of iMEFs due to the decrease in progenitor cell repopulation.

### iMEFs can effectively induce ectopic bone formation, chondrogenesis and adipogenesis upon BMP9 stimulation in vivo

**Figure 6:**
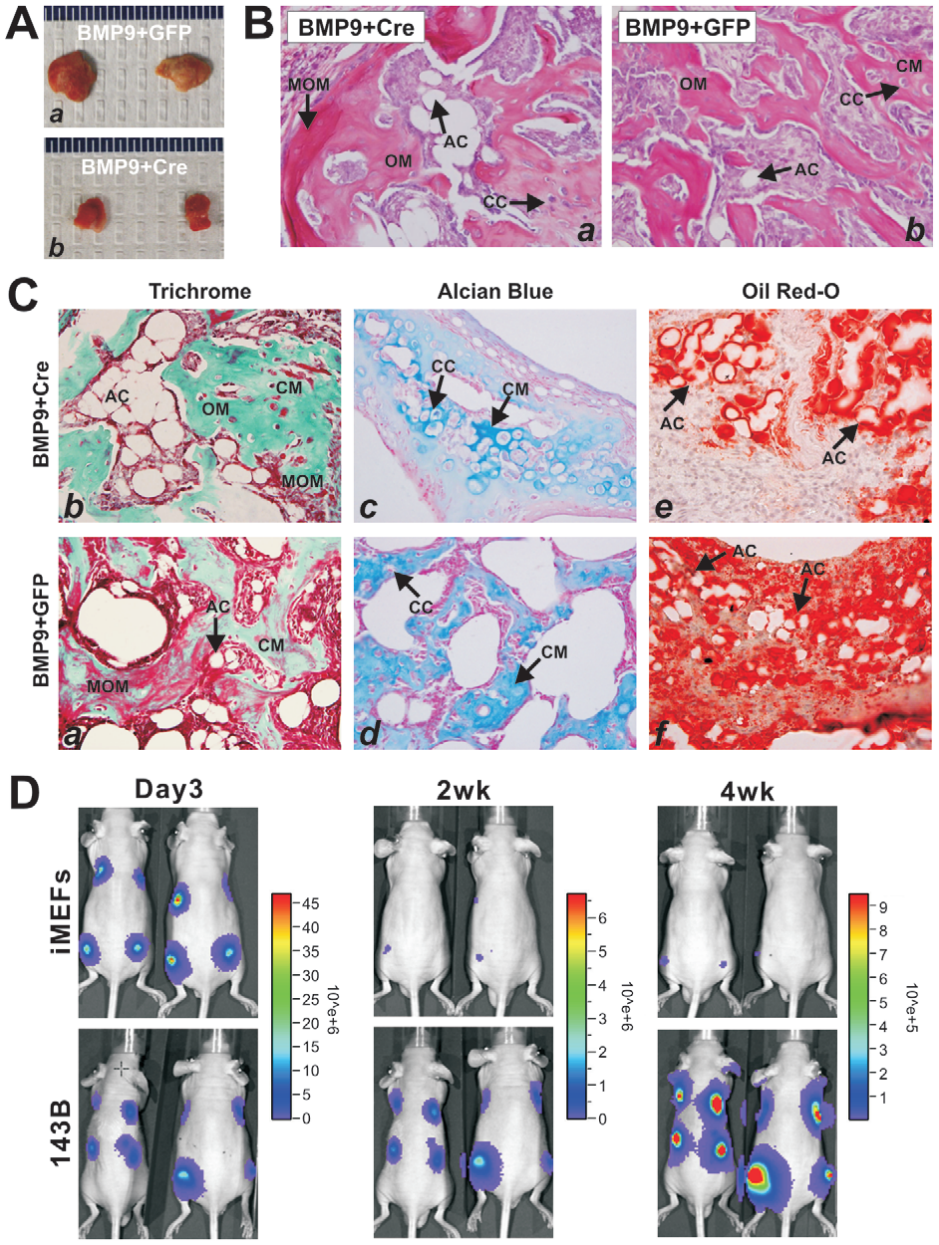
The iMEFs can effectively induce ectopic bone formation, chondrogenesis, and adipogenesis upon BMP9 stimulation *in vivo*; and yet the iMEFs are non-tumorigenic. (**A**) The iMEFs without the removal of SV40 T Ag form larger ectopic bone masses. Subconfluent iMEFs were co-infected with BMP9, GFP and/or Cre for 16 h. Cells were collected and injected into the flanks of athymic mice subcutaneously. Bony masses were retrieved from mice after 4 weeks. No masses were formed in the cells transduced with AdGFP or AdCre alone. (**B**) H & E staining. The retrieved bony masses were fixed, decalcified, and subjected to H & E staining. (**C**) Trichrome, Alcian Blue, and Oil Red-O staining of the retrieved samples. AC, adipocyte; CC, chondrocyte; CM, chondroid matrix; MOM, mineralized osteoid matrix; OM, osteoid matrix. (**D**) Potential tumorigenicity of the iMEFs. The iMEFs and human osteosarcoma line 143B cells were stably labeled with firefly luciferase and injected into the flanks of athymic nude mice (2×10^6^ cells/injection). At the indicated time points, animal were anesthetized with isoflurane and injected (i.p.) with D-Luciferin sodium salt at 100 mg/kg in 0.1 ml sterile saline. Bioluminescence imaging was conducted with the Xenogen IVIS 200 imaging system. The pseudoimages were obtained by superimposing the emitted light over the gray-scale photographs of the animals. Representative results are shown.

We next tested if the iMEFs would be able to undergo multi-lineage differentiation *in vivo*. Using the well-established stem cell implantation assay [29], [30], [32], [37]–[39], we co-transduced iMEFs with AdBMP9 and AdCre or AdGFP in culture and injected the cells subcutaneously into anthymic nude mice. Bony masses were retrieved from mice after 4 weeks (**Fig. 6A**), while no masses were formed in the cells transduced with AdGFP or AdCre alone (data not shown). Consistent with our *in vitro* results, the Cre-mediated removal of SV40 T antigen from iMEFs led to form smaller masses than that of GFP controls (**Fig. 6A**). H & E staining of the retrieved masses indicate that Cre-transduced iMEFs seemingly formed more mature and fully mineralized osteoid matrix (**Fig. 6B**, panel a) than that of the GFP control group (**Fig. 6B**, panel b). Nonetheless, adipocytes and chondrocytes were readily detected (**Fig. 6B**). Masson trichrome staining confirmed that both mineralized osteoid matrix and osteoid matrix were presented in Cre-treated and GFP-treated samples (**Fig. 6C**, panels a & b). The presence of chondrocytes and chondroid matrix was evident in both groups with Alcian Blue staining (**Fig. 6C**, panels c & d). Oil Red-O staining revealed that adipocytes and preadipocytes were abundantly found in both groups (**Fig. 6C**, panels e & f). Thus, the *in vivo* results strongly suggest that iMEFs may give rise to osteogenic, chondrogenic and adipogenic lineages. Furthermore, the immortalization of iMEFs can be reversed by Cre recombinase, which may lead to a lower proliferative activity and higher differentiation potential.

### iMEFs are not tumorigenic in athymic nude mice

One of the major concerns over using immortalized progenitor cells is their tumorigenic potential. Although SV40 large T antigen is not considered an oncogene in mammalian cells, it remains unclear if the T antigen would transform MEF progenitors and render tumorigenicity. Thus, we sought to determine if iMEFs were tumorigenic. Using the iMEFs stably tagged with firefly luciferase, we injected the cells into the flanks of athymic nude mice subcutaneously. We used human osteosarcoma line 143B as a positive control line. Cell proliferation at the injection sites was monitored by using a small animal whole body bioluminescence Xenogen IVIS 200 imaging system (**Fig. 6D**). We found that while initial signal was strong (**Fig. 6D**), the signal rapidly decreased at week 2 and became barely detectable at week 4. The signal was undetectable in the injected animals for up to 10 weeks and did not detect any tumor-like mass growth (data not shown). In contrast, the 143B cells grew rapidly and formed rather large xenograft tumors at week 4 (**Fig. 6D**). Taken together, these results have demonstrated that while iMEFs have an increased proliferative activity, they retain multipotency and are non-tumorigenic. It has been well-documented that SV40 T antigen-transformed cells are in general not tumorigenic [58]–[61]. Thus, these cells may be used as model systems for MSC biology and differentiation studies.

## Discussion

Primary MEFs serve as a rich source of mesenchymal progenitor cells, and have become a popular source of MSCs. Primary MEFs are also widely used as the feeder layer for culturing embryonic stem cells [62]. It is believed that MEFs can provide factors that enhance the proliferation and maintain the undifferentiated states of embryonic stem cells. Because the life span of primary MEFs is limited and isolation of primary MEFs is time-consuming and labor-intensive, there is always an essential need to develop MEFs with permanent growth features. Two major approaches have been developed to immortalize primary MEFs, including the serial passages of primary MEFs until they overcome their growth-crisis stage and the transformation of primary MEFs by overexpression of one or more oncogenes. The serial passage has been used to establish the BALB/3T3 cell line [63]. However, most of these established MEF lines were hypotetraploid [63]. Furthermore, it is unclear if these transformed cells retain multi-lineage differentiation potential. Numerous studies have been recently carried out to induce the pluripotency of fibroblasts and MEFs through reprogramming stem cell differentiation capability with a set of defined factors, such as Oct3/4, Sox9, Klf4, and c-Myc [54]–[57].

We investigate whether immortalized MEFs retain the multipotent properties of mesenchymal stem cells (MSCs) by taking advantage of a previously characterized reversible immortalization system using SV40 T antigen [28]. The reversible immortalization vector SSR #69 contains the hygromycin and SV40 T antigen expression cassette flanked with Cre/loxP sites [28]. Our results show that MEFs can be effectively immortalized with SV40 T antigen, and the proliferative activity of the iMEFs can be effectively reversed by Cre recombinase. The immortalized MEFs (iMEFs) express most of the MSC markers. Growth and differentiation factor BMP9 up-regulates the key lineage-specific regulators Runx2 (osteogenic), Sox9 (chondrogenic) and PPARγ2 (adipogenic), and induces osteogenic marker ALP activity and matrix mineralization in the iMEFs *in vitro*. The iMEFs are able to undergo terminal adipogenic differentiation upon BMP9 or PPARγ2 stimulation. Furthermore, *in vivo* stem cell implantation studies indicate that the iMEFs are able to bone, cartilage and adipose tissues upon BMP9 stimulation, whereas in the presence of Cre recombinase the ability of iMEFs to differentiate into these tissues is decreased largely due to the reduced expansion of progenitor population. Taken together, our results have demonstrated that the reversibly immortalized iMEFs not only maintain long-term cell proliferation but also retain the ability to differentiate into multiple lineages. The reversible SV40 T-mediated immortalization strategy should be used to establish stable cells from primary progenitors isolated from limited tissue sources, which would be critical for basic and translational studies.

The large T antigen encoded by simian virus 40 (SV40) plays essential roles in the infection of permissive cells, leading to production of progeny virions, and in the infection of nonpermissive cells, leading to malignant transformation [64], [65]. Primary MEFs are nonpermissive for SV40, and infection by wild-type SV40 leads to immortalization and transformation of a small percentage of infected cells. The ability of SV40 large T antigen to immortalize MEFs is largely dependent on its ability to complex with p53 [66]. Thus, SV40 T antigen has become one of the most commonly used gene to immortalize primary mammalian cells. In this report, we demonstrate that the iMEFs are not tumorigenic at least within the duration of observation. The Cre-mediated reversal of the immortalization phenotypes should further enhance the safety profile of this strategy. Nonetheless, it is noteworthy that the Cre-mediated excision of SV40 T antigen is not footprintless as a significant portion of the retroviral vector, such as LTRs and packaging signal, remains integrated in the host genome.

Traditional cell immortalization and transformation strategies have been widely used in cancer research [67]. Those transformation approaches usually involves in overexpression of oncogenes and/or inactivation of tumor suppressor genes. Many other genes have been used to immortalize primary cells. The commonly used oncogenes may include k-ras, c-myc, CDK4, cyclin D1, Bmi-1, and HPV 16 E6/E7, to name a few, while the frequently inactivated tumor suppressor gens are p53, Rb, and p16^INK^, etc. Unlike these oncogenes, it has been well-documented that SV40 T antigen-transformed cells are in general not tumorigenic [58]–[61].

Another commonly used gene for immortalization is telomerase (TERT) [68]. The telomerase complex maintains telomere length, which is required for an unlimited cellular proliferation. Telomerase is low or absent in normal human somatic cells. Telomerase expression is high in stem cells but reduced upon differentiation. Restoring telomerase activity in normal somatic cells can indefinitely prolong cellular life span, which may be associated with the acquisition of characteristics typical of cellular transformation [69]. Ectopic expression of the catalytic subunit of mouse telomerase (mTERT) confers a growth advantage to primary MEFsand facilitates their spontaneous immortalization by targeting the TGFβ pathway [70]. A recent study indicated that ectopic expression of mouse telomerase catalytic subunit (mTERT) does not affect embryonic stem (ES) cell proliferation or differentiation *in vitro*, but protects ES cells against cell death during differentiation [71]. Nonetheless, the efficiency and differentiation potential of TERT-immortalized MSCs need to thoroughly investigated.

In summary, to overcome the challenges in maintaining sufficient MSCs for *in vitro* and *in vivo* studies, as well as for possible clinical applications, we demonstrate that MEFs can be efficiently immortalized by SV40 T antigen. The reversibly immortalized iMEFs exhibit high proliferative activity and maintain long-term cell proliferation, which can be reversed by introducing Cre recombinase. The immortalized MEFs express most of the MSC markers and retain multipotent differentiation potential as they can differentiate into osteogenic, chondrogenic and adipogenic lineages under appropriate differentiating conditions both *in vitro* and *in vivo*. Furthermore, the immortalized MEFs are apparently not tumorigenic when subcutaneously injected into athymic nude mice. Thus, the reversible SV40 T antigen-mediated immortalization is an efficient and safe approach to establishing long-term cell culture of primary mesenchymal progenitors for basic and translational research. This reversible immortalization strategy should be a valuable tool for studying primary progenitors which are isolated from limited tissue availability.
