# Correction: Conditionally Immortalized Mouse Embryonic Fibroblasts Retain Proliferative Activity without Compromising Multipotent Differentiation Potential

**DOI:** 10.1371/annotation/2f1c6442-0ecb-4a33-9534-aae89b5fbbc1

**Published:** 2013-01-15

**Authors:** Enyi Huang, Yang Bi, Wei Jiang, Xiaoji Luo, Ke Yang, Jian-Li Gao, Yanhong Gao, Qing Luo, Qiong Shi, Stephanie H. Kim, Xing Liu, Mi Li, Ning Hu, Hong Liu, Jing Cui, Wenwen Zhang, Ruidong Li, Xiang Chen, Jikun Shen, Yuhan Kong, Jiye Zhang, Jinhua Wang, Jinyong Luo, Bai-Cheng He, Huicong Wang, Russell R. Reid, Hue H. Luu, Rex C. Haydon, Li Yang, Tong-Chuan He

The following information was missing from the Funding section: The reported work was also supported in part by a research grant from the Natural Science Foundation of China (No. 81100309 to YB). 

